# Characterization of Mutational Status, Spheroid Formation, and Drug Response of a New Genomically-Stable Human Ovarian Clear Cell Carcinoma Cell Line, 105C

**DOI:** 10.3390/cells9112408

**Published:** 2020-11-03

**Authors:** Bart Kolendowski, Yudith Ramos Valdes, Hal Hirte, Hiroaki Itamochi, Wonjae Lee, Mark Carey, Trevor G. Shepherd, Gabriel E. DiMattia

**Affiliations:** 1 Mary & John Knight Translational Ovarian Cancer Research Unit, London Regional Cancer Program, London, ON N6A 5W9, Canada; bkolendo@uwo.ca (B.K.); yudithramos@yahoo.es (Y.R.V.); wlee377@uwo.ca (W.L.); tshephe6@uwo.ca (T.G.S.); 2 Lawson Health Research Institute, London, ON N6C 2R5, Canada; 3 Juravinski Cancer Centre, Hamilton, ON L8V 5C2, Canada; hirteh@hhsc.ca; 4 Department of Obstetrics and Gynecology, Tottori University School of Medicine, Nishi-cho, Yonago-shi, Tottori-ken 683-0826, Japan; itamochi@hotmail.com; 5 Michael G. DeGroote School of Medicine, McMaster University, Hamilton, ON L8P 1H6, Canada; 6 Department of Obstetrics & Gynaecology, University of British Columbia, Vancouver, BC V6Z 2K8, Canada; mark.carey@ubc.ca; 7 Department of Oncology, Western University, London, ON N6A 5W9, Canada; 8 Department of Anatomy & Cell Biology, Western University, London, ON N6A 3K7, Canada; 9 Department of Obstetrics & Gynaecology, Western University, London, ON N6H 5W9, Canada; 10 Department of Biochemistry, Western University, London, ON N6A 5C1, Canada

**Keywords:** clear cell, ovarian, cancer, cell line, mTOR inhibitor, spheroid, epithelial ovarian cancer, OCCC

## Abstract

Ovarian clear cell carcinoma (OCCC) is a rare subtype of gynecological cancer for which well-characterized and authenticated model systems are scarce. We provide an extensive characterization of ‘105C’, a cell line generated from an adenocarcinoma of the clear cell histotype using targeted next-generation sequencing, cytogenetic microarrays, along with analyses of AKT/mTOR signaling. We report that that the 105C cell line is a bona fide OCCC cell line, carrying PIK3CA, PTEN, and ARID1A gene mutations, consistent with OCCC, yet maintain a stable genome as reflected by low copy number variation. Unlike KOC-7c, TOV-21G, and RMG-V OCCC lines also mutated for the above genes, the 105C cells do not carry mutations in mismatch repair genes. Importantly, we show that 105C cells exhibit greater resistance to mTOR inhibition and carboplatin treatment compared to 9 other OCCC cell lines in 3D spheroid cultures. This resistance may be attributed to 105C cells remaining dormant in suspension culture which surprisingly, contrasts with several other OCCC lines which continue to proliferate in long-term suspension culture. 105C cells survive xenotransplantation but do not proliferate and metastasize. Collectively, we show that the 105C OCCC cell line exhibits unique properties useful for the pre-clinical investigation of OCCC pathobiology.

## 1. Introduction

Ovarian clear cell cancer (OCCC) is a histotype of epithelial ovarian cancer (EOC) that accounts for 5–10% of all ovarian cancers diagnosed [1,2]. Histologically, OCCC presents as cells with clear cytoplasm due to an accumulation of glycogen which is then lost when the tissue is treated with formalin. Elegant studies have shown that endometriotic tissue contiguous with an OCCC lesion contains the same gene mutations characteristic of OCCC. This means that endometriotic tissue is the origin of clear cell cancer of the ovary and is a result of retrograde deposition of this tissue on surfaces within the peritoneal cavity especially the ovary [3,4,5,6]. 

OCCC patients make up to 26% stage I/II ovarian cancer patients and show a good outcome [2,7,8,9]. However, when OCCC patients present with late stage disease, the outcome is poor because the disease is resistant to conventional epithelial ovarian cancer chemotherapeutics or quickly becomes refractory [10,11,12]. While the need to develop treatments for OCCC is pressing, understanding the reasons why chemotherapy fails and identifying therapeutic targets has been difficult due in part to the lack of robust model systems, which in turn is because a very limited number of OCCC cell lines are available. Franklin et al. 2018 provided a comprehensive list of 25 OCCC cell lines [13] but only 12 of these cell lines are obtainable from commercial repositories. Anglesio and co-workers [14] have also provided a detailed molecular analysis of human ovarian cancer cell lines indicating 7 lines from the CCLE list of 49 human ovarian cancer cell lines as bona fide OCCC lines. Domcke et al. [15] also reported a comprehensive molecular analysis of human ovarian cancer cell lines calling into question the origin of some of the most popular human ovarian cancer cell lines cited in the literature. Therefore, there is a clear need to provide the research community with a variety of OCCC lines which encompass the complete spectrum of genomic characteristics reflecting the scope of human OCCC. 

In this study, we characterize the 105C cell line that was originally derived from the ascites of an ovarian cancer patient and identified as “adenocarcinoma of the ovary with clear cell differentiation” [16]. This histopathological classification of ovarian tumours has been addressed by Takenaka and co-workers [17] using targeted DNA sequencing of potential driver genes coupled with immunohistochemistry demonstrating that extensive characterization of the tumour genome can clarify ovarian cancer subtype. Our goal was to perform a similar broad characterization of the 105C cell line to provide strong evidence classifying it as a new OCCC cell line. We used next-generation sequencing of the 105C cell line to interrogate 161 cancer related genes. We compared the 105C line to nine other OCCC cell lines (TOV-21G, KOC-7c, OVMANA, OVTOKO, SMOV-2, TU-OC-1, RMG-I, ES-2, and OVSAYO) regarding mTOR pathway activity, epithelial and mesenchymal markers, copy number alterations, and multi-cellular cluster or spheroid formation in suspension culture. We discovered that several OCCC cell lines proliferate in suspension culture. Given that a high percentage of OCCC tumours carry mutations which result in hyperactivation of the AKT/mTOR pathway, we examined the ability of an mTOR inhibitor, AZD-8055 to effect cell viability. We examined the susceptibility of OCCC lines to AZD-8055 when cultured as a proliferating monolayer or whilst in suspension as multi-cellular clusters as would occur in patient ascites. The ability of AZD-8055 to specifically inhibit the reattachment of OCCC cell line spheroids formed in suspension culture was measured as an indicator of the capacity of an mTOR inhibitor to reduce spheroid adhesion. The viability of OCCC cell lines in suspension culture over an extended time period was assessed to determine spheroid morphology, proliferative potential and longterm viability. In addition, we tested the ability of the 105C line to generate tumours in immunocompromised mouse models. We show that the 105C cell line carries gene mutations found in the OCCC histotype and that it, like three other OCCC cell lines analyzed, showed a high level of genome stability. In response to mTOR inhibition the 105C line responded differently from the other nine lines tested with it showing relative drug resistance depending on culture condition. 

## 2. Materials and Methods 

### 2.1. Cell Culture and Reagents

Cell lines were cultured in Dulbecco’s modified Eagle medium/Ham’s F12 (Wisent) and supplemented with 10% FBS (Wisent). The 105C cell line (originally referred to as SCHM-1) was obtained from Dr. Hal Hirte (McMaster University, Hamilton, ON, Canada). The KOC-7c, RMG-I, SMOV-2, OVMANA, OVTOKO, TU-OC-1, and OVSAYO cell lines were provided by Dr. Hiroaki Itamochi (Iwate Medical University, Iwate-ken, Japan). TOV-21G cell line was provided by Dr. Anne-Marie Mes-Masson (University of Montréal, Montreal, QC, Canada). ES-2 cell line was kindly provided by Dr. Barbara Vanderhyden (University of Ottawa, Ottawa, ON, Canada).

Cells were maintained in at 37 °C and a CO_2_ concentration of 5%. For experiments requiring spheroid formation ultra-low attachment plates (Corning, NY, USA) were used. At the time of experimentation, cell lines were positively identified using short tandem repeat (STR) analysis performed at the Centre for Applied Genomics (TCAG) in Toronto, Ontario, Canada and cross referenced to STR fingerprints in the CLASTR 1.4.4 database (https://web.expasy.org/cellosaurus-str-search/) (Supplementary Table S1). Cells in monolayer were maintained by allowing cells to reach ~90% confluency at which point the media was aspirated and cells were washed with phosphate buffered saline (PBS) and exposed to 2 mL of trypsin/EDTA until cells detached from plate. Cells were resuspended in 10 mL of media and then split onto new plates at a minimum of a 1:3 dilution. ECM Gel from Engelbreth–Holm–Swarm murine sarcoma (no. E1270) and Collagenase, from Clostridium histolyticum (no. C6885), were both purchased from MilliporeSigma (Oakville, ON, Canada). 

### 2.2. Mutation Profiling

#### 2.2.1. Mutation Assay

To determine the mutational profile of the 105C cell line we used the Oncomine Comprehensive v3 assay (Thermo Fisher Scientific, Mississauga, ON, Canada). Genomic DNA obtained from 105C cell line was sent to the Sunnybrook Research Institute Genomics Core Facility in Toronto, Ontario, Canada where the assay was performed according to the manufacturer’s protocol. The data was analyzed by the Sunnybrook Genomics Core Facility using their standard filter (Amplicon locations: Exonic, Splice sites, 5′ UTR, 3′ UTR; Nucleotide variant (MNV), Single nucleotide variant (SNV), Insertion or deletion (InDel) and Copy number variant (CNV); Variant effects: frameshift insertion, non-frameshift insertion, frame shift deletion, non-frameshift deletion, stopless, missense, nonsense; UCSC Common SNPs: filtered out; 1000 Genomes-Minor Allele Frequency (MAF): >0.01 filtered out; 5000 Exomes Global MAF: >0.01 filtered out; CNV Confidence Range: >10; *p*-value (data quality): >0.01 filtered out). In addition to the Sunnybrook Genomics Core Facility Standard Filter, we removed any hits where the number of sequence reads mutated were less than 30% of total reads obtained. Data accession number GSE160571.

#### 2.2.2. Comparison with Publicly Available Datasets

To compare and contrast the mutational profile of the 105C cell line to other OCCC cell lines we downloaded the cell line specific mutational information from the CCLE at the DepMap repository [18]. Next-generation characterization of the Cancer Cell Line Encyclopedia. Nature 569, 503–508 (2019) and (DepMap, Broad (2019): DepMap 19Q3 Public. figshare. Dataset doi:10.6084/m9.figshare.9201770.v2). Using a custom Python script, we extracted all cell types where “lineage” was “ovary”, “primary_or_metastasis” was “Primary”, and sub_subtype was “ovary_clear_cell” or “ovary_endometrioid”. We listed all mutations that were classified as anything other than ‘silent” as being “Mutated” for the purpose of our analysis (i.e., “Missense_Mutation”, “Nonsense_Mutation”, “In_Frame_Del”, ‘splice_Site”, “Frame_Shift_Del”, “Frame_Shift_Ins”, and ‘start_Codon_SNP”). Finally, we limited our analysis to genes which were identified as being mutated in the 105C cell line. The custom Python script generated for this analysis is available upon request. 

To compare our data with tumor samples, we obtained data from the AACR Project Genomics Evidence Neoplasia Information Exchange (GENIE) dataset. To identify samples of interest, the data selector at http://genie.cbioportal.org/was first used to identified sample IDs that were classified as “Ovarian Cancer” (under the “Cancer Type” header). These IDs were then used to generate a “User-defined Case List” from the GENIE Cohort v6.1-public dataset. A custom Python script was used to extract mutation data from the dataset and limit analysis to genes mutated in the 105C cell line. 

### 2.3. Whole Exome Analysis

To determine mutation status for SMOV-2 and OVSAYO, we obtained raw reads for these two samples from the SRA (SRA: ERP010768), uploaded them to the Galaxy web platform, and analyzed using tools available at the public server, usegalaxy.org. The workflow, specific parameters used for a particular tool, and specific details related to identifying variants from SRA were obtained from (https://training.galaxyproject.org/training-material/topics/variant-analysis/). Briefly, reads obtained from SRA were mapped to hg19 using the Burrows–Wheeler Aligner (BWA) (Galaxy Version 0.7.17.4) [19] and PCR duplicates were removed using RmDup (Galaxy Version 2.0.1). Variant were identified using the Bayesian genetic variant detector FreeBayes (Galaxy Version 1.3.1) [20], normalized using (bcftools) and then annotated and filtered using SnpEff eff (Galaxy Version 4.3 + T.galaxy) [21]. Data was visualized using Integrative Genomics Viewer (IGV) version 2.4.11 (UC San Diego, La Jolla, CA and the Broad Institute of MIT and Harvard, Cambridge, MA, USA).

### 2.4. Genome Copy Number Determination 

Copy number information for OCCC cell lines not publicly and readily available was determined using the CytoScan HD assay and performed at the Centre for Applied Genomics (TCAG) in Toronto, Ontario, Canada. The CytoScan HD assay was carried out according to the standard protocol recommended by the manufacturer (reference file “cytoscanhd_array.na32.3.v1”). Segmented data was obtained from Chromosome Analysis Suite (Applied Biosystems/Thermo Fisher Scientific, Mississauga, ON, Canada, version 4.0.0.385) and files were converted to seg format (hg19). Publicly available segmented copy-number profiles in the .seg file format (hg19) were obtained from the CCLE (https://portals.broadinstitute.org/ccle/data:“CCLE_copynumber_2013-12-03.seg.txt”). To obtain data from tumor samples, we downloaded segmented copy number data from the GENIE database (release 4.1-public) and filtered it for OCCC samples for which copy number was assayed. Percentage of genome was calculated by dividing the number of regions in the genome that were altered by the effective genome size (hg 19). The data were then sorted from lowest to greatest percentage of genome altered. Data was visualized using Integrative Genomics Viewer (IGV) version 2.4.11. Data accession number GSE160571.

### 2.5. Determining IC_50_ of AZD-8055 in Monolayer and Spheroid Cultured OCCC Cell Lines

#### 2.5.1. AZD-8055 Treatment and IC_50_ Curve Generation 

Seeding concentration was determined for each cell line by plating cells at different densities and measuring the response to alamarBlue reagent (Thermo Fisher Scientific, Mississauga, ON, Canada) on day 5 to ensure cells were within the dynamic range of the alamarBlue reaction. Cells were seeded in triplicate at their optimized density in a 96-well format on both tissue culture plastic to ensure monolayer formation as well as ultra-low attachment plates (ULA, Corning) to induce spheroid formation. At 48 h after initial plating, cells were treated with AZD-8055 (MedChemExpress, Monmouth Junction, NJ, USA, HY-10422) at the listed concentration. At 72 h post-treatment (96-h post seeding), cells were treated with alamarBlue, which uses the cells reductive capacity to allow a quantitative measure of cell viability, and absorbance was measured using a Wallac Victor2 multi-label counter. To determine the IC_50_ of AZD-8055 for the OCCC cell lines, the alamarBlue response signal was normalized to the signal obtained for cells treated with control (DMSO), represented by 100% viability, while those treated with the highest concentration of the drug correspond to 0% viability. The AZD-8055 doses were then transformed using dose = Log (dose) and finally the normalized and transformed data was analyzed using the non-linear regression (curve fit) in GraphPad Prism 6 for Windows (Version 6.01) analysis package, using the “log(inhibitor) vs. normalized response” equation. IC_50_ values and R^2^ values were outputted directly from GraphPad Prism 6.

#### 2.5.2. OCCC Cell Line Spheroid Reattachment in the Presence of AZD-8055 

To determine the response of OCCC cell line spheroids to mTOR inhibition upon reattachment to tissue culture plasticware, we followed two different approaches using five OCCC cell lines: 105C, OVMANA, TOV-21G, RMG-I, and KOC-7c. First, cells were seeded to 24-well ULA plates for 48 h and then treated for 72 h with DMSO or AZD-8055 at the determined spheroid IC_50_ concentration for each cell line. Spheroids were then transferred to allow attachment to 24-well regular tissue culture plastic (Sarstedt, Montreal, QC, Canada) for 72 h. To assess the effect of AZD-8055 treatment during attachment to tissue culture plasticware, cells were seeded to 24-well ULA plates for 5 days. Spheroids were then treated at the time of reattachment to 24-well tissue culture dishes (Sarstedt, Montreal, QC, Canada) with DMSO or the AZD-8055 IC_50_ concentration determined for monolayer culture for each cell line, respectively. In both instances, viability was assessed 72 h after spheroid reattachment using alarmarBlue per the manufacturer’s protocol. 

### 2.6. Carboplatin Treatment of OCCC Cell Line Spheroids

To determine the sensitivity of OCCC cell line spheroids to carboplatin (Accord Healthcare, Kirkland, Quebec) we selected three OCCC cell lines (KOC-7c, RMG-I, and TOV-21G) that showed a proliferative phenotype while in suspension, and two OCCC cell lines (105C and OVMANA) which were quiescent while in suspension. KOC-7c, RMG-I, and TOV-21G cell lines were seeded at 50,000 cells per well. 105C and OVMANA cell lines were seeded at 100,000 cells per well. Cells were culture in 24-well ULA plates (Corning) for 48 h and then treated with 50 μM or 100 μM of carboplatin. Untreated cells were used as controls. Three days after treatment, spheroids were collected and trypsinized at 37 °C for 10 min to dissociate the clusters for cell counting. Trypan blue exclusion cell counting was performed using a TC20 Automated Cell Counter (Bio-Rad, Mississauga, ON, Canada).

### 2.7. 105C Cell Line Proliferation Assay (Doubling Time)

To determine cell line doubling time cells were seeded at 10,000 cells per well in 48-well plates (Corning, NY, USA) using four technical replicates. Cell proliferation was monitored using IncuCyte™ real-time imaging technology (Sartorius, Oakville, ON, Canada). Cells were imaged every 3 h for up to 111 h and confluence was measured using IncuCyte™ software. Doubling time was calculated by fitting an exponential growth curve to the confluence-over-time data using the nonlinear regression (curve fit) package from GraphPad Prism 6.01 (GraphPad Software, San Diego, CA, USA).

### 2.8. OCCC Cell Line Spheroid Culture Viability Assay

We seeded 100,000 cells in triplicate in 24-well ULA plates (Corning, NY, USA) in 1 mL of DMEM: F12 and 10% FBS. On day 7 and day 15 of culture, 1 mL of fresh media was added to each well. To determine cell number, the contents of each well were collected and spun down at 1500 rpm for 3 min. The cells pellet was washed with PBS and resuspended in 50 μL of 0.25% Trypsin (Wisent) and incubated for 15 min at 37 °C to dissociate the clusters of cells. Trypan blue exclusion cell counting was performed using a TC20 Automated Cell Counter (Bio-Rad, Mississauga, ON, Canada).

### 2.9. Western Blotting

For cells cultured as adherent monolayers, whole cell extracts were obtained by scraping cells into radioimmunoprecipitation assay (RIPA) buffer supplemented with phosphatase inhibitors. Lysates were vortexed thoroughly and incubated on ice for 30 min, prior to centrifugation at 14,000× *g* for 30 min. The pellet was discarded and the supernatant protein concentration was determined using Bradford assay Protein Dye Reagent (Bio-Rad, Mississauga, ON, Canada). For protein lysates from spheroids, cell suspensions were collected from ULA plates and centrifuged at 2400 rpm for three minutes. The cell pellet was washed with ice cold PBS and placed in modified RIPA lysis buffer for 30 min on ice and processed as described above to generate a protein lysate. 50 µg of protein was loaded and subjected to electrophoresis using Bolt 4–12% Bis-Tris Plus polyacrylamide gels (Invitrogen) according to the manufacturer’s protocol. The Bio-Rad “Trans-Blot Turbo” kit and system was used for western blot transfer following the manufacturer’s instructions. All PDVF membrane blocking and washing steps were done in Tris-buffered saline with Tween 20 (20 mM Tris, 150 mM NaCl and 0.1% Tween 20) and with either 5% skim milk or 5% BSA (depending on antibody requirements). All antibodies were used at recommended concentrations from the manufacturer. Mouse or rabbit secondary antibodies were used to probe primary antibodies for 1 h at room temperature, followed by 3 × 10 min washes with TBST and finally incubation with Immobilon Classico Western HRP Substrate or Immubilon Forte Western HRP Substrate (MilliporeSigma (Oakville, ON, Canada)) for 5 min. Western blot imaging and signal quantification was performed using a Bio-Rad ChemiDoc XRS+ system. 

### 2.10. Antibodies 

Antibodies against PIK3CA (#4255S), PTEN (D5G7) (#5384S), phospho-4E (#2855S), 4E-BP1(#9452S), phospho-Akt (Ser473) (#9271S), AKT (#9272S), phospho-p70 S6K (#9234S), p70 S6K (#2708S), E-Cadherin (24E10) (#3195S), N-Cadherin (D4R1H) (#13116S), Vimentin (D21H3) (#5741S), and EpCAM (D9S3P) (#14452) were purchased from Cell Signaling (Whitby, ON, Canada). Antibodies against Vinculin (#V9264), Actin (#A2066), CK-7 (Clone OV-TL 12/30) (#MAB3554), and HNF1-beta (clone 12A5.1) (#MABE971) were obtained from MilliporeSigma (Oakville, ON, Canada). Antibody against ARID1A/BAF250 (#A301-041A-M) was purchased from Bethyl Laboratories (Montgomery, Texas, United States). HRP-conjugated antibodies against mouse IgG (#NA931) and rabbit IgG (#NA934) were procured from GE Healthcare Life Sciences (Baie d’Urfe, QC, Canada). 

### 2.11. Statistical Analysis

All statistical analyses were performed using GraphPad Prism 6.01 (GraphPad Software, San Diego, CA, USA). The results were assessed using a two-tailed Student’s *t*-test or a two-way ANOVA with Tukey’s multiple comparisons test., a *p*-value of less than 0.05 was considered statistically significant.

### 2.12. 105C Cell Line Murine Xenografting Studies

Xenograft animal experiments were approved by Institutional Animal Care and Use Committee of the University of Western Ontario (2015-65) and carried out in accordance with approved guidelines. *NOD.CB17-Prkdc/NCrCrl* (NOD/SCID) female mice from Charles River Laboratories (#394) (8–10 weeks old) were inoculated by intraperitoneal injection with 1 × 10^6^ cells in 150 µL of PBS. 

*NOD-scid IL2Rgammanull* (NSG) female mice from Jackson Laboratory (#005557) and *NOD.CB17-Prkdc/NCrCrl* (NOD/SCID) female mice from Charles River Laboratories (Laval, Quebec, Canada; catalogue #394) (8–10 weeks old) were injected subcutaneously and bilaterally with 1 × 10^7^ cells in 150 µL of PBS mixed with ECM Gel from Engelbreth–Holm–Swarm murine sarcoma at a 1:1 ratio. Mice were sacrificed 85 and 140-days post-injection respectively and examined for tumor formation. Small white nodules were excised and digested in 30 µg/µL of collagenase from *Clostridium histolyticum* (C6885; MilliporeSigma, Oakville, ON, Canada) at 37 °C for one hour and the resultant cells placed in culture with Dulbecco’s modified Eagle medium/Ham’s F12 and supplemented with 10% FBS to recover resident cancer cells. 

## 3. Results

### 3.1. Gene Mutational Analysis

**Figure 1:**
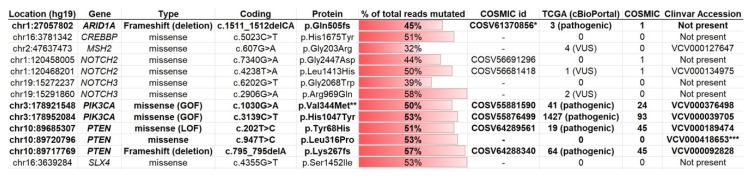
Genes mutated in the 105C cell line. Targeted sequencing of 161 cancer-driver genes was performed using the Oncomine v3 assay in the 105C cell line. The horizontal red bar provides a measure of the mutated gene allele frequency with the exact value in the middle of each bar. The gene mutations known to be deleterious to protein function are in bolded text. Mutations were filtered to exclude common SNPs and Minor Allele Frequency (MAF) > 0.01 and % of total reads mutated < 30%. *: This COSMIC id for *ARID1A* describes a frameshift mutation that is similar, but not identical to, the mutation found in 105C. **: Identified as hotspot. ***: Identified as ‘likely pathogenic’ in macrocephaly/autism syndrome and Cowden syndrome 1. Type: “GOF”: Known to result in a gain of function: “LOF”: Known to result in loss of function. TCGA: indicates number of unique cases reported with the mutation; VUS: variant of unknown significance. COSMIC: indicates number of unique cases reported with the mutation in the COSMIC database. Clinvar: indicates whether the specific mutation has been annotated in this database. “VUS”: Variant of Unknown Significance.

To establish the mutational profile of 105C cell line we performed targeted next-generation sequencing of 161 genes using the Oncomine Comprehensive v3 assay. Analysis revealed that the 105C cell line contains nonsynonymous mutations in *ARID1A, PIK3CA*, and *PTEN*, resulting in loss of expression, gain-of-function, and loss-of-function, respectively (Figure 1). In *ARID1A*, we identified a loss of the CA residues encoding Q505 (c.1511_1512delCA) which is predicted to result in a frameshift and the formation of a TAA terminator codon 117 amino acids downstream. We also identified three different *PTEN* mutations, two missense mutations and a deletion causing a frameshift. The heterozygous *PTEN* p.Tyr68His and p.Lys267fs mutations have both been previously annotated in the COSMIC database and are described as deactivating mutations. In vitro phosphoinositide studies carried out by Han and colleagues [22] showed that the p.Tyr68His, caused by a T to C transition, results in an enzyme which completely lacks phosphatase activity and at the time of this writing, eight tumors were recorded in the cBioPortal database carrying this amino acid change in PTEN. The PTEN p.Lys267fs mutation resulted from loss of an A-residue within a stretch of six at the carboxy terminus of exon 7. The p.Leu316Pro found in the 105C *PTEN* gene is listed as a variant of unknown significance by the Clinical Genome Resource (https://www.genome.gov/Funded-Programs-Projects/ClinGen-Clinical-Genome-Resource) and this mutation was not found in cBioPortal. The p.Val344Met PIK3CA change is a recurrent hotspot listed as a likely oncogenic mutation in cBioPortal. Bourgon and coworkers, interpreting the crystal structure of PIK3CA suggest that a change at V344 is likely to reduce the interaction between the PIK3R1 and PIK3CA thus allowing greater enzymatic activity [23]. The p.His1047Tyr change found in the 105C PIK3CA is the most frequently mutated residue in the protein and is well established as an activating mutation [24]. These findings are revealing as mutations in these genes are most often observed in OCCC and are almost entirely absent in HGSC [14,15,25,26]. Additionally, no mutations were found in TP53, which is ubiquitously mutated in HGSC [15]. Taken together, these data support the 105C cell line generated from the OCCC histotype.

**Figure 2:**
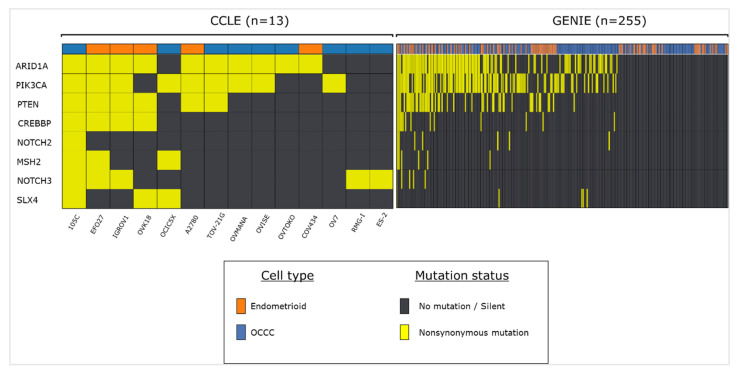
Frequency of 105C cell line-related mutations in endometrioid and OCCC tumour samples and cell lines. Using publicly available datasets we assessed the mutational status of genes that we previously characterized as mutated in the 105C cell line. Cell line data was obtained using CCLE database and tumor sample information was obtained from the GENIE database.

We also compared the gene mutation profile of the 105C cell line to publicly available mutation data from both tumour tissue, obtained from ovarian cancer patients in the Genomics Evidence Neoplasia Information Exchange (GENIE) database, as well as ovarian cancer cell lines, obtained from the Cancer Cell Line Database (CCLE) (Figure 2). Not surprisingly, we find that the 105C mutational profile matches that of many OCCC cell lines and human tumor samples with respect to the *ARID1A*, *PIK3CA*, and *PTEN* genes. Additionally, some of the other mutations that we identified in our screen of the 105C cell line (e.g., *CREBBP*) appear in other OCCC cell lines and tumour samples, suggesting that these genes may play a yet unidentified role in a subset of these cancers.

### 3.2. ARID1A Expression Analysis

**Figure 3:**
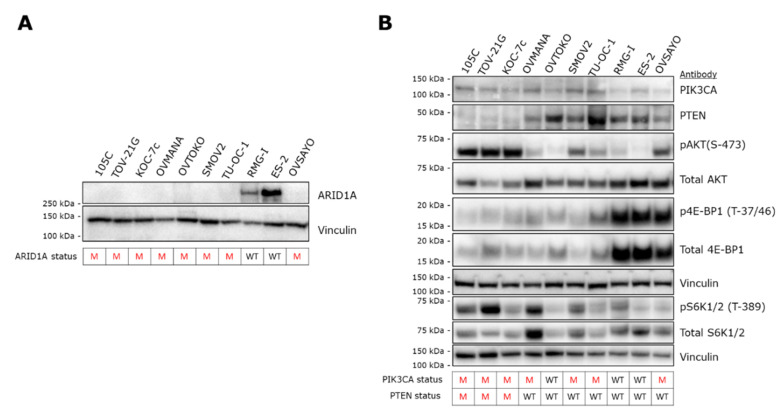
Expression of gene products of NGS-detected mutated genes in OCCC cell lines. (**A**) Western blot analysis to assess the expression of ARID1A revealed no detectable signal from 105C cells as well as all the other OCCC cell lines that are mutated for *ARID1A*. In contrast, RMG-I and ES-2 cell lines, wildtype for *ARID1A*, produced an ARID1A band of the expected M.W. Vinculin was used as loading control. *ARID1A* mutation status is shown below the blots. M: mutant; WT: wildtype. (**B**) To determine the downstream effects of the *PIK3CA* and *PTEN* mutations in 105C cells in relation to 10 other OCCC cell lines, we performed western blot using antibodies targeting components of the AKT signaling pathway. Notably, cell lines containing both *PIK3CA* and *PTEN* mutations (105C, TOV-21G, and KOC-7c) show the highest level of pAKT (S-473). Vinculin was used as loading control. The *PIK3CA* and *PTEN* mutation status is shown below the blots. Densitometric quantification of western blot signals is provided in Supplementary Figure S1. M: mutant; WT: wildtype.

To supplement our mutational analysis, we determined the steady-state level of proteins that were identified as mutated in the 105C cell line (Figure 3). Genomic DNA sequence analysis revealed a 2bp deletion in the 105C *ARID1A* gene leading to a frameshift and therefore, a likely loss of expression of the full-length protein. We performed western blot analysis using an antibody specific to the *ARID1A* gene product to compare the level of ARID1A in 105C cells to a subset of OCCC cell lines. ARID1A was not detectable in 105C cells or any of the OCCC lines known to carry a mutated ARID1A gene. In contrast, protein lysates from cell lines previously documented (Supplementary Table S2) to carry wildtype *ARID1A* (ES-2, RMG-I), were positive for expression (Figure 3A). While our mutational analysis revealed that the *ARID1A* mutation is heterozygous, an accompanying total loss of detectable ARID1A protein is not without precedent; previous studies have made parallel observations indicating that mutations in both *ARID1A* alleles are found in only ~30% of OCCC tumors and 73% of tumors that are heterozygous for inactivating mutations in *ARID1A* show complete loss of ARID1A histological staining [3,27,28].

### 3.3. AKT/mTOR Pathway

To characterize the effects of the above described mutations on PIK3CA signaling, we isolated whole cell extracts and immunoblotted for levels of PIK3CA, PTEN as well as downstream components of the PIK3CA pathway in the 105C cell line and compared the steady-state level of these markers to nine other OCCC lines whose *PIK3CA* and *PTEN* mutational status is known (Figure 3B). Similar to the other cell lines that contain *PIK3CA* and *PTEN* mutations (Supplementary Table S2), the 105C cell line shows a relatively high level of phosphorylation of AKT (S-473), an indicator of active PIK3CA signaling. Interestingly, S6K1/2 a downstream marker of mTOR signaling, also shows an increase in phosphorylation (relative to total S6K1/2 levels) in 105C and TOV21G, both which possess mutated *PIK3CA* and *PTEN* (Supplementary Table S1). All other cell lines which do not harbor both mutations did not show an obvious increase in phosphorylation of S70 S6K relative to total S70 S6K protein levels. Interrogation of 4E-BP1 levels revealed no relationship to any particular gene mutational profile.

### 3.4. Phenotypic Characterization of the 105C Cell Line

**Figure 4:**
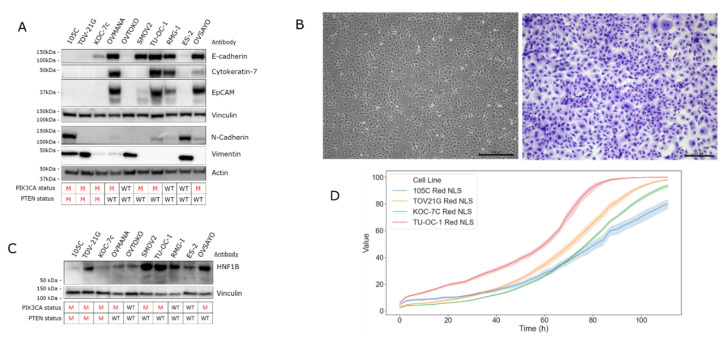
Molecular characterization of the 105C cell line. (**A**) Western blot analysis to assess the expression of mesenchymal and epithelial associated gene products was performed on the 105C cell line and nine other OCCC lines. The 105C cell line did not demonstrate detectable epithelial markers, but showed expression of the mesenchymal markers, N-cadherin and vimentin whereas OVMANA, SMOV-2, TU-OC-1, RMG-I, OVSAYO, and OVISE showed detectable expression of epithelial markers. (**B**) Imaging and staining of 105C cell line using Hema 3 reveals a cobblestone appearance. (**C**) Western blot of HNF1B abundance in the 105C cell line relative to OCCC cell lines. siRNA knockdown experiment of HNF1B mRNA was performed to verify western blot bands that corresponded to HNF1B. Vinculin and actin were used as loading controls. The *PIK3CA* and *PTEN* mutation status is shown below the blots. M: mutant; WT: wildtype. (**D**) Confluency of various OCCC cell lines was measured every 3 h for 111 h using the IncuCyte™ platform.

To characterize the epithelial/mesenchymal characteristics of the 105C cell line we interrogated whole cell lysates of 10 OCCC lines, using a panel of antibodies specific to mesenchymal or epithelial markers (Figure 4A). OVMANA, TU-OC-1, OVSAYO, and RMG-I showed abundant steady-state levels of epithelial markers E-cadherin, cytokeratin-7, and EpCAM and very low or undetectable levels of vimentin and N-cadherin. Whereas 105C, ES-2, OVTOKO, and TOV-21G cell lines displayed low to undetectable levels of canonical epithelial markers and easily detectable mesenchymal marker expression. The KOC-7c and SMOV-2 lines demonstrate E-cadherin expression and very low or undetectable expression of the other markers. As expected, this panel of OCCC lines is heterogeneous regarding epithelial phenotype with the 105C line distinctly mesenchymal. Despite this, the 105C cell line, when grown in monolayer, exhibits a cobblestone-like appearance, more generally associated with epithelial cell types (Figure 4B). 

In addition to interrogating epithelial and mesenchymal markers we also determined the levels of hepatocyte nuclear factor1B (HNF1B) which is expressed in most OCCC samples while relatively undetectable in the other subtypes, making it a reliable marker of OCCC (Figure 4C) [17,29]. Importantly, because endometrioid and OCCC histotypes share many common characteristics (likely due to a similar cell of origin) HNF1B is one of the few molecular markers that can be used to distinguish between the two subtypes [29,30]. Western blot analysis using an antibody specific for HNF1B revealed that all 10 OCCC cell lines generate a HNF1B signal of varying intensity, with SMOV-2, TU-OC-1, and ES-2 cell lines showing the strongest signal intensity and the 105C line producing the weakest signal. To determine the doubling rate of the 105C cell line relative to other cell lines we used the Incucyte ZOOM system and monitored proliferation for up to 111 h (Figure 4D). We found the 105C cell line doubling time at 31.3 h was slower relative to other OCCC cell lines: TOV-21G (25.5 h), KOC-7c (24.0 h), and TU-OC-I (26.4 h) (Supplementary Table S2). 

### 3.5. Whole-Genome Copy Number Variation Analysis

**Figure 5:**
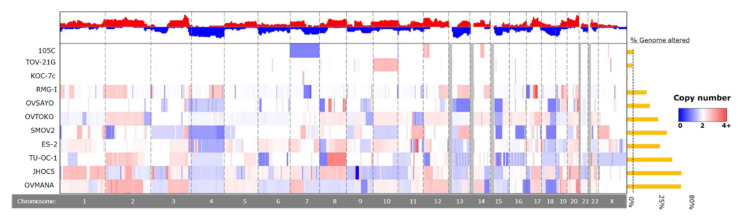
Chromosomal copy number alterations in 105C cell line compared to other OCCC cell lines. To identify copy number alterations in OCCC cell lines we assayed 105C, KOC-7c, SMOV-2, TU-OC-I, and OVSAYO using Cytoscan HD and obtained CNA publicly available data for TOV-21G, RMG-I, OVTOKO, ES-2, OVISE, OVMANA, and JHOC5 cell lines from the CCLE. Red indicates DNA amplification and blue indicates DNA deletion, with intensity of the color indicating degree of change, with darker colors reflecting higher levels of amplification/deletion. Chromosome identity is provided in the bottom row. Greyed-out areas are genomic regions left out of the analysis due to low coverage.

**Figure 6:**
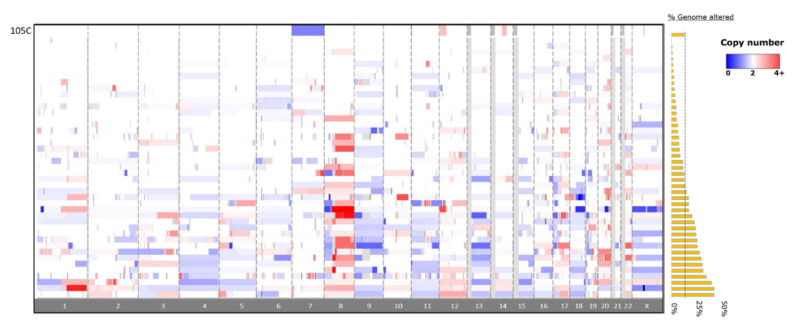
Copy number alterations in the 105C OCCC cell-line compared to patient tumors from the GENIE database. Copy number alterations in the 105C line are focal in nature and are primarily restricted to chromosome 7, 12, and 14 with an overall 8% change in genome integrity. The OCCC tumor CNA data (*n* = 43) indicates a wide variety of chromosomal changes showing between 0.1% and 46.7% of genome altered. Red indicates DNA amplification and blue indicates DNA deletion. Greyed-out areas are genomic regions left out of analysis due to low coverage.

To identify significant gene deletions and amplifications that might contribute to the 105C phenotype and determine the degree of genomic instability describing the 105C cell line relative to other OCCC lines, we performed copy number analysis using the Cytoscan HD platform on 105C, KOC-7c, SMOV-2, TU-OC-1, and OVSAYO lines. This data along with CCLE and GENIE copy-number data allowed us to compare the 105C line to 11 OCCC cell lines as well as patient tumour samples (Figure 5 and Figure 6, Supplementary Table S2). Our analysis revealed that, similar to the KOC-7c and TOV-21G cell lines, the 105C cell line is characterized by a highly stable genome with the majority of the alterations seen as a broad shallow deletion spanning chromosome 7. This is consistent with previous findings that large copy number alterations indicative of significant genomic instability in OCCC and mucinous tumors occur less often than high grade serous ovarian cancer [31]. 

### 3.6. Spheroid Morphology

**Figure 7:**
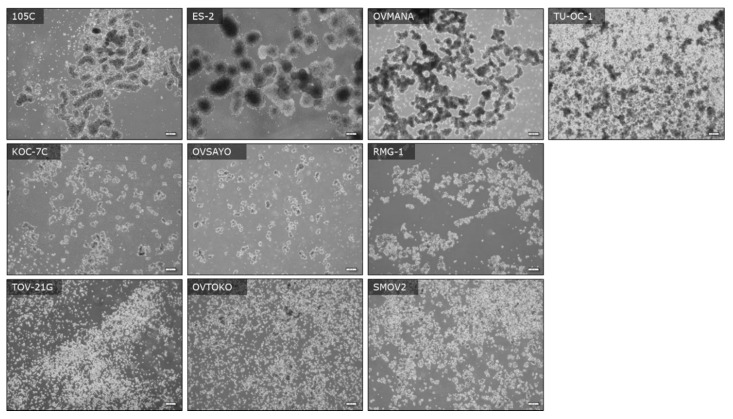
Images of OCCC cell lines seeded to ULA plates showing the morphology of autonomously formed multicellular clusters (spheroids) and presence of single and dead cells at day-3 of culture. Cell lines were seeded to six-well ULA plates at a density of 500,000 cells per well and images were captured with an inverted Olympus microscope at the 72 h timepoint. Scale bars represent 200 μM.

It is well-established that tumors resident in the peritoneal cavity can produce ascites and that tumour cells released into the ascites can subsequently migrate to other surfaces resulting in the formation of secondary tumour sites [32]. Therefore, OCCC cell viability in suspension culture is relevant to metastasis as the cancer cells should survive anoikis to seed secondary sites of tumor growth. To examine the morphological characteristics and viability of the 105C cell line relative to the other OCCC lines we seeded them to ultra-low attachment (ULA) surfaces to promote multicellular cluster formation or spheroids. We find that after 3 days, 105C cells form tight spheroid clusters with defined boundaries, similar to those of ES-2, OVMANA, and TU-OC-1 cell lines. In contrast, the remaining OCCC cell lines (TOV-21G, KOC-7c, OVTOKO, SMOV-2, RMG-I, OVSAYO) can be grouped into those that that form loosely clustered clumps and those that exist primarily as single cells in suspension (Figure 7). 

**Figure 8:**
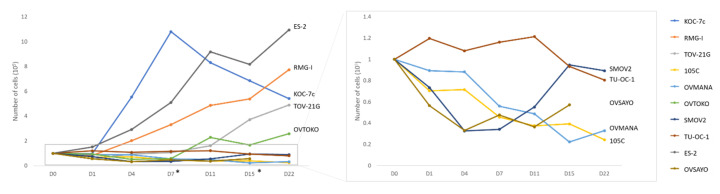
Viability of OCCC cell lines cultured as spheroids in suspension. 100,000 cells were seeded in triplicate in 24-well ULA plates and cultured for up to 22 days. Trypan blue exclusion counting was performed at each time point and the total number of viable cells per well is shown for each cell line. While we found that a subset of OCCC cell lines (ES-2, RMG-I, KOC-7c, TOV-21G) proliferate in suspension culture, the majority of the OCCC cell lines including the 105C cells remained cytostatic or lost viability when maintained in suspension culture over the period of the assay. *: Fresh media added on D7 and D15.

To determine whether the OCCC cell lines including 105C cells remain in a quiescent state during spheroid formation, cells were seeded to 24-well ULA plates and cultured for up to 22 days. Trypan blue exclusion counting was performed at different time points. Interestingly, we found that a subset of OCCC cell lines (ES-2, RMG-I, KOC-7c, TOV-21G) continue to proliferate in suspension culture while the majority of the OCCC cell lines including the 105C cells showed significant loss of cell viability when cultured in suspension (Figure 8). The KOC-7c cell line showed a loss of cell viability after day 7 of spheroid culture. This correlated with a significant loss of media buffering capacity in KOC-7c cultures after day 7 indicative of high metabolic activity which reduced cell viability over time. 

### 3.7. AKT/mTOR Signaling in OCCC Cell Lines Cultured as Adherent Monolayer Versus Spheroid Suspension Culture

**Figure 9:**
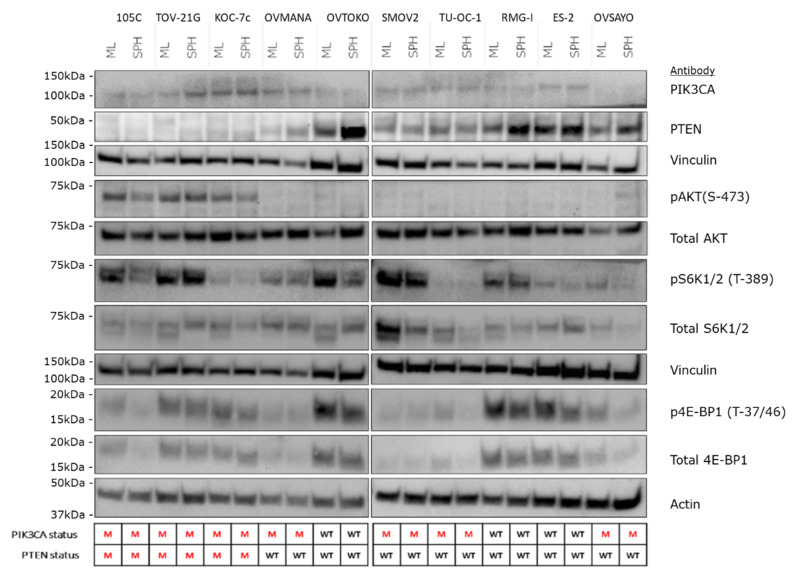
Western blot analysis of AKT/mTOR signaling in OCCC cell lines cultured as adherent versus 72 h spheroid suspension culture. Major changes in the abundance of AKT/mTOR signaling components across all OCCC lines was not observed when they were cultured as spheroids in suspension. Changes in the relative levels of specific components of the pathway were observed as focal changes in specific cell lines. Vinculin and actin were used as loading controls. The *PIK3CA* and *PTEN* mutation status is shown below the blots. M: mutant; WT: wildtype.

Previously, we have characterized the changes in AKT signaling when HGSC cell lines were cultured in suspension as spheroids versus proliferating monolayer cultures and found that most lines became quiescent in spheroid culture [33]. Our data showing that some OCCC lines proliferate in suspension culture suggested that AKT/mTOR signaling may be maintained in spheroids. Moreover, to determine whether mutations in the AKT/mTOR pathway (*PIK3CA* and *PTEN*) impact signaling we performed western blot analyses on proliferating monolayer cells and cells cultured in suspension, using antibodies targeting various components of the pathway (Figure 9). Interestingly, the steady-state signal for pAKT was strongest in cell lines (105C, KOC-7c, and TOV-21G) with both mutated *PTEN* and *PIK3CA* under both monolayer and spheroid conditions, while OCCC lines wild-type for both genes or those lines with only *PIK3CA* mutated showed a relatively weak or non-detectable signal for pAKT. In contrast, phosphorylation of S6K and 4E-BP1does not appear to be correlated with the mutation status of *PIK3CA* or *PTEN* in the cell lines tested regardless of culture condition. 

### 3.8. OCCC Cell Line Sensitivity to the mTORC1/2 Inhibitor, AZD-8055 

**Figure 10:**
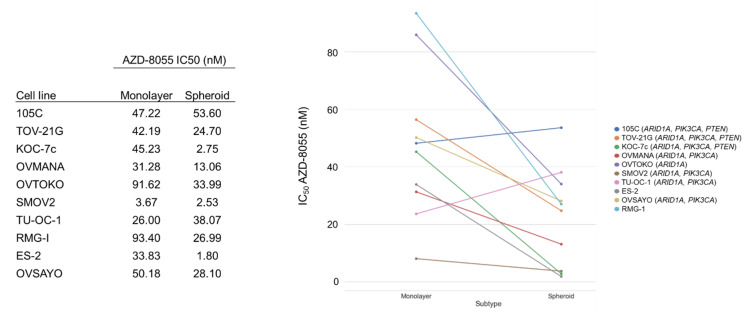
OCCC cell line sensitivity to the mTORC1/2 inhibitor, AZD-8055, when cultured as monolayer versus spheroid suspensions. The cell lines were cultured as monolayers or spheroids in suspension for 48 h and then treated for 72 h with a range of AZD-8055 concentrations to determine the IC_50_ for each line. AlamarBlue was used to assess viability. 105C cells did not show an increase in sensitivity to mTOR inhibition in suspension cultures as occurred for most of the OCCC cell lines tested. Genes in brackets are mutated in the cell line.

Due to the prevalence of *PIK3CA* and *PTEN* mutations in OCCC, inhibition of the mTOR pathway has been postulated as a potential therapeutic target. Recent work by Caumanns et al., has demonstrated that indeed, OCCC cell lines are exceptionally sensitive to AZD-8055, an mTORC1/2 inhibitor, which was able to disrupt OCCC tumor growth in vivo [26]. While the sensitivity of cells grown in monolayer is clear, whether this sensitivity extends to OCCC spheroids remains to be elucidated. Previous work has also shown that *PIK3CA* activating mutations alone do not correlate with the sensitivity of a cell line to mTOR inhibition, but the impact of co-mutations in *PIK3CA* and *PTEN*, which correlate with elevated pAKT levels, has not been assessed [34,35,36,37]. To determine the sensitivity of OCCC spheroids and if *PIK3CA/PTEN* mutations play a role in mediating this sensitivity, 10 OCCC cell lines were cultured as monolayers or spheroids in suspension for 48 h and then treated for 72 h with a range of AZD-8055 concentrations to determine the IC_50_ for each line for each culture condition (Figure 10 and Supplementary Figure S2). To ensure that mTOR pathway inhibition was maintained over the course of the experiment, the 105C cell line was treated with AZD-8055 and phosphorylation of AKT was monitored over 4 days by western blot which demonstrated complete loss of the pAKT signal at 1 µM and significantly reduced pAKT at the IC_50_ (Supplementary Figure S3). Remarkably, we find that OCCC cell lines generally exhibit increased sensitivity to mTOR inhibition in suspension cultures, compared to monolayer. No obvious relationship was found between the sensitivity to AZD-8055 and the mutation status of *PIK3CA* and/or *PTEN*, and unexpectedly, we found that a subset of OCCC cell lines proliferate while in suspension and it is this phenotype that may sensitize them to AZD-8055-mediated mTOR inhibition. Interestingly, the 105C line is one of the few cell lines that did not show an obvious increase in sensitivity to mTOR inhibition in suspension cultures compared to those cultured as monolayers.

**Figure 11:**
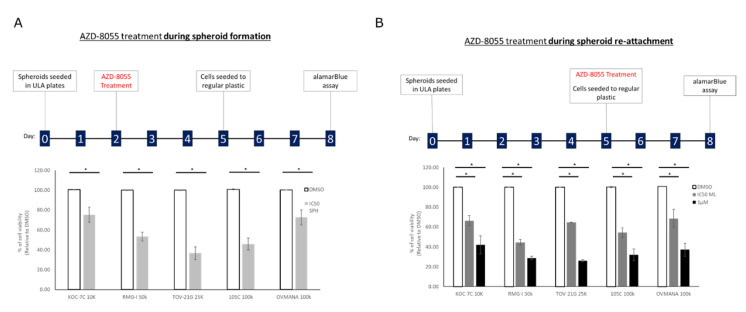
AZD-8055 reduces OCCC cell line viability during spheroid formation and spheroid reattachment. (**A**) Cells were seeded into 24-well ULA plates for 48 h and then treated with DMSO, the AZD-8055 spheroid IC_50_ for each cell line respectively or 1 μM AZD-8055 for 72 h. Spheroids were then reattached for 72 h into 24-well regular tissue culture plastic at which point viability was assessed by alamarBlue assay. (**B**) Cells were seeded into 24-well ULA plates for 5 days. Spheroids were then treated at the time of reattachment into 24-well regular tissue culture plastic with DMSO, the AZD-8055 monolayer IC_50_ for each cell line respectively or 1 μM AZD-8055. 72 h after reattachment viability was assessed by alamarBlue assay. Viability data was normalized to DMSO-treated controls set to 100%. A one-way ANOVA with Tukey’s multiple comparisons test was performed to determine statistical significance for each cell line (*, *p* < 0.05) (*n* = 3).

Spheroids are considered metastatic units that eventually will adhere to the peritoneum to continue to invade at secondary sites [38,39]. To gain further insight into the response of spheroids to mTOR inhibition, we performed a double-dose experiment where cells were treated with AZD-8055 at their respective IC_50_, while in suspension culture or just at the time of spheroid reattachment to regular tissue culture plasticware (Figure 11). In this way, we expected to discern whether AZD-8055 would affect cell viability primarily while cultured in suspension and if AZD-8055 might selectively affect the ability of cells in suspension to then attach to an adherent surface. For the KOC-7c cell line treated only during suspension culture and reattached without drug, we observed a 25% drop in viability whereas when spheroids treated with AZD-8055 only at the time of spheroid attachment to tissue culture plastic displayed only an 6% reduction in cell viability. When KOC-7c spheroid cells were reattached at the AZD-8055 IC_50_ for monolayer culture, the reduction in cell viability was 37%. RMG-I cells treated only during suspension spheroid culture or only at the time of spheroid reattachment showed a similar (47%) reduction in cell viability. AZD-8055 caused a large drop in cell viability (64%) with the TOV-21G cell line in suspension culture and much less so when treated at the time of spheroid reattachment. The OVMANA cell line in suspension culture showed a 28% loss in cell viability in the presence of AZD-8055 and a similar (32%) decrease when treated with the IC_50_ concentration of AZD-8055 for monolayer cells. The 105C cell line in suspension culture was highly sensitive to AZD-8055 dropping cell viability by 55% and when treated at the time of spheroid reattachment, 46% of the cells remained viable at the IC_50_ determined for spheroid suspension culture. It is also notable that the viability of all cell lines was more adversely affected by AZD-8055 treatment while cells were in suspension culture relative to when treated at the time of spheroid reattachment. This was most evident at the 1uM dose of AZD-8055 used as the positive control for AZD-8055 efficacy. 

**Figure 12:**
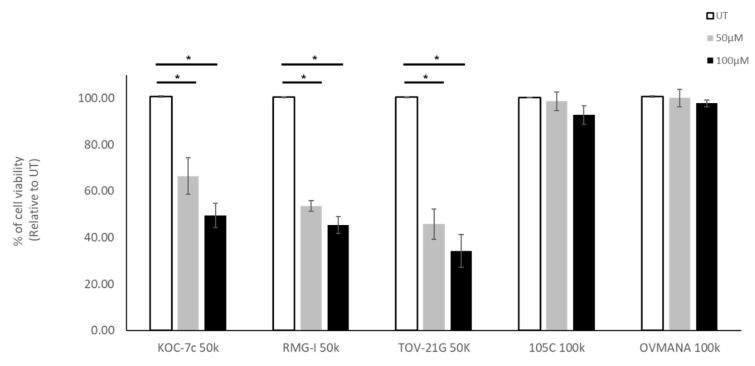
Effect of carboplatin treatment on OCCC cell lines in spheroid culture. Cells were seeded into 24-well ULA plates for 48 h and then treated with 50 μM or 100 μM of Carboplatin. Untreated cells were use as control cultures (UT). Trypan Blue Exclusion cell counting was performed 72 h after treatment. Viability data was normalized to untreated controls set to 100%. A one-way ANOVA with Tukey’s multiple comparisons test were performed to identify statistically significant differences in cell viability for each cell line (*, *p* < 0.05) (*n* = 4).

In addition to mTOR inhibition we also treated OCCC cell lines with carboplatin, a therapeutic often used to treat EOC. To determine whether proliferation rate played a role in sensitivity to carboplatin, we selected cells that proliferate while in suspension (KOC-7c, RMG-I, and TOV-21G) and cell lines that are static in suspension culture (105C and OVMANA). Notably cell lines which proliferate when in suspension culture, showed increased sensitivity to carboplatin treatment when cultured as spheroids. The OVMANA and 105C cell lines, which do not proliferate in suspension culture, were however, not affected by carboplatin treatment at either concentration (Figure 12). Clearly, the ability of these OCCC cell lines to proliferate in suspension culture impacts their sensitivity to carboplatin in contrast to the effects of AZD-8055. 

### 3.9. 105C Cell Line Xenograft Studies 

To determine whether the 105C cell line would form xenograft tumors in immunocompromised mouse strains, we performed intraperitoneal and subcutaneous injection into NOD/SCID and *NOD-scid IL2Rgammanull* (NSG) female mice. Tumour formation was not observed in any of the mice after intraperitoneal injection. We detected small white nodules at the sites of subcutaneous injection which were excised for cell culture and histology. Nodules from each mouse strain were pooled, digested with collagenase to generate a single cell suspension, and placed in cell culture. Cells dissociated from the nodules grew in culture and the recovered cells were identified by STR analysis (Supplementary Table S1) as the 105C line. 

## 4. Discussion

### 4.1. Genetic and Molecular Characterization

We have performed an extensive characterization of the 105C cell line to determine the epithelial ovarian cancer histotype from which it was derived and provide this cell line to the research community. As stated previously, the 105C line was originally reported by Hirte and coworker in 1994, as derived from a mixed tumour histology noted as Stage IIC papillary serous adenocarcinoma of the ovary with clear cell differentiation [16]. To establish the subtype from which this cell line was derived we performed a wide-array of studies.

Gene mutational analysis provided the first indication that the 105C cells were derived from an OCCC given the presence of the *ARID1A* mutation along with loss of function mutation in *PTEN* and an activating mutation in *PIK3CA*. This constellation of mutations is commonly seen in OCCC (Supplementary Table S2). While our NGS data revealed that the 105C *ARID1A* mutation is heterozygous, western blot analysis revealed that the protein product is not detectable suggesting a complete loss in expression. This observation is not entirely surprising as others have also observed a similar loss of expression with a corresponding mutation in one of the alleles [3,27,28]. An analysis of *ARID1A* mutations found in cBioPortal (1036 mutations), at the time of this writing, showed that a stomach adenocarcinoma (TCGA-FP-8631-01) contained the same *ARID1A* mutation found in the 105C line.

The combination of three mutations in *PTEN* resulted in the loss of detectable PTEN by western blot in 105C cell line protein lysates like KOC-7c and TOV-21G cell lines which also carry *PTEN* mutations. We also performed an extensive analysis of the literature and using the CCLE database in combination with reports providing detailed gene mutational data [14,26,40], we conclude that of the 27 reported OCCC cell lines [13] only three carry the constellation of mutations that typify OCCC tumors, namely the RMG-V, TOV-21G, and KOC-7c lines each carry mutations in *ARID1A*, *PTEN*, and *PIK3CA* [41]. It is also notable that the RMG-V, KOC-7c, and TOV-21G lines are considered hypermutated given that they carry mutations or methylated loci in DNA mismatch repair genes [40,41] whereas the 105C line does not. Moreover, Shen and co-workers [42] showed that ARID1A and DNA mismatch repair proteins interact, meaning that the loss of both proteins could lead to a hypermutated phenotype. This further distinguishes the 105C cell line as it does not carry debilitating mutations in mismatch repair proteins. Global 105C gene expression analysis indicates no loss of mismatch repair gene expression (data not shown). Endometrioid carcinomas, which originate from the same tissue as OCCC, have been shown to frequently harbor mutations in genes such as *CTNNB1* (42% of samples), *KMT2D* (31%), *KMT2B* (19%), and *PIK3R1* (19%) [43]. Interestingly, the RMG-V cell line harbors mutations in the *KMT2A*, *KMT2D*, *CTNNA1*, and *CTNNB1* genes [41]; KOC-7c carries mutations in *KMT2A*, *KMT2D* [41], and TOV-21G also carries pathogenic mutations in *KMT2C* and *KMT2D* genes [44,45] and lacks MLH1 expression due to gene methylation [41,42].

The epithelial to mesenchymal transition in cells of epithelial origin has been identified as a hallmark of cancer and specifically implicated as promoting dissemination and invasiveness during tumorigenesis [46]. In the context of EOC, markers of EMT have been shown to be associated with increased peritoneal dissemination, resistance to chemotherapy, progression-free survival, and overall survival [47,48,49]. To establish the phenotypic characteristics of the 105C cell line as a potential model of EMT in OCCC, we examined the steady-state levels of five canonical epithelial or mesenchymal markers relative to 9 other OCCC cell lines. The 105C cell line grouped with the TOV-21G, KOC-7c, OVTOKO, and ES-2 cell lines which exhibited a mesenchymal phenotype based on the lack of expression of epithelial markers. The OVMANA, SMOV-2 TU-OC-I, RMG-I, and OVSAYO lines displayed a distinctly epithelial phenotype. The loss of E-cadherin and concomitant gain of vimentin expression is indicative of cells having undergone an epithelial to mesenchymal transition, which is often associated with more aggressive forms of cancer [50,51,52,53]. It is also notable that the expression of these phenotypic markers does not correlate with a distinct gene mutational profile in OCCC cell lines, the morphology of the spheroids they generate in suspension culture or whether the cell line proliferates in suspension culture. 

The expression HNF1B has been identified as a marker that is expressed preferentially in OCCC and is silenced in the other subtypes [17,29,30]. Western blot for HNF1B across the 10 OCCC lines revealed strong signals in lysates from SMOV-2, TU-OC-1, RMG-I, and ES-2 lines while a low steady state level of HNF1B was observed in 105C cells along with the other OCCC lines tested. Our data adds to the evidence at the immunohistochemical [17,29,30,54] and PCR [55] level for HNF1B as a marker of OCCC. 

We also examined HNF1B gene expression using the cBioPortal platform in EOC cell lines and found that the five OCCC cell lines (JHOC-5, RMG-I, TOV-21G, OVTOKO, OVMANA) show the highest HNF1B mRNA levels amongst the 47 EOC cell lines analyzed in the CCLE. However, robust HNF1B expression is not an exclusive property of OCCC lines as indicated by Wiedmann et al., as they showed OCCC cell line HNF1B levels similar to that of the SKOV3 cell line [56]. Additionally, Ince and co-workers demonstrated by western blot that of the OCCC lines they established only four showed detectable HNF1B protein expression [57]. These data confirmed that HNF1B expression in cell lines is highly variable even within the OCCC histotype and the low level of HNF1B expression in 105C cells is not unexpected given the variability seen across OCCC cell lines by other investigators. 

### 4.2. Copy-Number Analysis

Previous studies have highlighted that EOC subtypes differ with respect to genomic stability: HGSC exhibit a high level of genomic instability due to mutated DNA repair pathways which results in a large proportion of their genome undergoing copy number variation, whereas the OCCC and endometrioid subtypes are generally much more stable and show far less copy number alteration [43,58]. We assessed the level of genomic variation in eleven OCCC cell lines and found that they exhibited a wide-range of copy number variation (ranging from 0.88% to 73.5%, (Supplementary Table S2) with three cell lines (KOC-7c, TOV-21G, and 105C) possessing remarkably unaltered genomes (0.88%, 7.46%, 8.12% of genome altered, respectively)) which puts them into the CIN-low arm of OCCC defined by Uehara and co-workers [59]. It is of note that the three OCCC cell lines which carry *PTEN* and *PIK3CA* mutations show the lowest levels of genomic instability. Numerous reports have indicated that loss of PTEN activity is mechanistically linked to loss of genomic stability; therefore, it is surprising that the 105C, KOC-7c, and TOV-21G cell lines show the lowest level of genomic instability relative to the other OCCC cell lines [60,61,62,63]. We also compared the 105C cell line copy number profile to the GENIE dataset and found that OCCC tumour samples contain less in the way of CNA, on average, than what is observed in OCCC cell lines. This can be explained by the fact that copy-number analysis of tissues is complicated by the presence of tumor stroma as well non-tumour tissue obfuscating the ‘true’ copy-number change, which is often higher [64]. 

### 4.3. mTOR pathway Inhibition and Carboplatin Treatment

Dysregulation of the AKT-mTOR pathway has been implicated in the development and progression of many different types of cancers [65,66]. Given that hyperactivation of this pathway has been observed in both early- and late-stage OCCCs (76% and 96% respectively) [67], it is reasonable to suggest that mTOR pathway inhibition may be a viable therapeutic target [26,68]. While studies testing the effects of mTOR inhibition have been reported using OCCC cell lines in monolayer culture [26,68,69,70], the effects of mTOR inhibition on OCCC cells maintained as spheroids have not been reported. The use of spheroid cell culture is relevant as these cell clusters represent 3D avascular structures which can be the conduits of metastatic disease for cancers originating in the abdomen [32]. We examined the importance of a constitutively active mTOR pathway to the survival these cells in suspension culture, we treated cells with the mTORC1/2 inhibitor AZD-8055 and measured their viability in both monolayer and in suspension. We first examined the viability of OCCC cell lines in suspension culture over time and made the unexpected observation that several lines (RMG-I, TOV-21G, KOC-7c, ES-2) proliferate in suspension culture. This was surprising as previous reports have characterized EOC and other cancer cell spheroids as quiescent and non-proliferative [33,71,72,73,74]. We found most OCCC lines showed a dramatic increase in sensitivity to AZD-8055 as measured by cell viability when maintained in spheroid culture. However, the 105C and TU-OC-1 cell lines showed the opposite trend. Additionally, an obvious relationship between sensitivity to AZD-8055 and the mutation status of *PIK3CA* and *PTEN* was not observed as the TOV-21G and KOC-7c exhibited increased sensitivity while the 105C line showed decreased AZD-8055 sensitivity when maintained in spheroid culture. We also asked whether the efficiency by which OCCC cell line spheroids can reattach to tissue culture plastic changed if only exposed to AZD-8055 while in spheroid culture or when treated just the time of spheroid reattachment to tissue culture plastic. We found that the OCCC cell line spheroids exhibited similar sensitivity to AZD-8055 regardless of when they were treated meaning that an initial exposure while OCCC spheroids where in suspension inhibited their ability to reattach efficiently. The viability of OCCC cell line spheroids treated once they were transferred to tissue culture plastic did not change significantly meaning that inhibition of mTORC1/2 signaling in OCCC cell line spheroids caused cell death in suspension culture and inhibited the ability of OCCC spheroids to attach to an adherent surface. Hence, in our studies, mTORC1/2 activity in OCCC cell lines is not only important to actively proliferating monolayer cells but can also significantly reduce their ability to remain viable in suspension culture. Our data suggests that mTORC1/2 inhibitors could reduce metastatic spread of OCCC as AZD-8055 can effectively kill cells in spheroid form and then reduce their ability to interact with an adherent substratum. 

These results suggest that further investigation into the mechanisms governing the suspension proliferation rate of cell lines may be helpful in not only identifying markers that can be informative regarding the extent to which patients might benefit from chemotherapeutic intervention, but also identifying new targets of therapeutic potential.

### 4.4. Murine Tumour Xenograft Model

We performed xenografting experiments with the 105C cell line using various strains of immunocompromised murine models which did not result in robust tumour formation. Interestingly, 105C cells were isolated from the site of injection. Hence, 105C cells can take up residence in murine tissue to form small nodules. The mutation profile, genome copy number variation and other properties did not predict the xenograft result given that TOV-21G, RMG-V, and KOC-7c cell lines readily produce xenograft tumors in mice with a similar mutation profile and degree of genome copy number variation to the 105C line [75,76,77]. We examined the literature and found that other OCCC cell lines, including NUOC-1 [78], TAYA [79], and TU-OC-II [80] did not form xenograft tumors. From an experimental design perspective, cell lines which do not form robust tumours in immunocompromised mouse models could be useful in assessing the tumour promoting properties of specific gene products as reported for the human breast cell line MCF10A [81,82,83]. 

In conclusion, we have identified and extensively characterized the 105C cell line as derived from OCCC despite the initial tumor pathology suggesting a mixed tumour type. The 105C line is one of the few carrying the prototypical OCCC mutation profile and yet is weakly tumourigenic in a xenograft model. The addition of the readily available 105C line to the compendium of OCCC cell lines provides researchers with a well-characterized model system for preclinical studies focused on the discovery and testing of novel therapeutic agents to treat OCCC.

## Supplementary Materials

The following are available online at https://www.mdpi.com/2073-4409/9/11/2408/s1, Figure S1: Densitometry measurements of total and phosphorylated protein levels for components of the mTOR pathway. Figure S2: Dose-response curves for the mTORC1/2 inhibitor, AZD8055. Figure S3: Four day time-course examining the effects of AZD-8055 on pAKT (S-473) levels on 105C monolayer cultures. Table S1: STR data for OCCC cell lines used in this study. Table S2: Molecular and phenotypic characteristics of the OCCC cell lines used in this work.
