# Supplementary material for: Characterization of Mutational Status, Spheroid Formation, and Drug Response of a New Genomically-Stable Human Ovarian Clear Cell Carcinoma Cell Line, 105C
Source: Cells. 2020 Nov 3;9(11):2408. doi: 10.3390/cells9112408 (PMC7693681; doi:10.3390/cells9112408)
Supplement: Supplementary file 1 [file cells-09-02408-s001.zip › revised Supplementary Table S1 - STR data for OCCC cell lines used in this study.docx]

|  | **ES-2** | **RMG-I** | **OVMANA** | **OVISE** | **OVSAYO** | **TOV-21G** | **OVTOKO** | **KOC-7c** | **SMOV2** | **TU-OC-I** | **105C** | **105C-T** |
| --- | --- | --- | --- | --- | --- | --- | --- | --- | --- | --- | --- | --- |
| **Amelogenin** | **X, X** | **X, X** | **X, X** | **X, X** | **X, X** | **X, X** | **X, X** | **X, X** | **X, X** | **X, X** | **X, X** | **X, X** |
| CSF1PO | 10, 10 | 10, 10 | 12, 13 | 9, 11 | 12, 12 | 13, 14, 15 | 12, 12 | 11, 12 | 10, 12 | 11, 12 | 13, 14 | 13, 14 |
| D13S317 | 11, 11 | 12, 12 | 8, 9 | 11, 12 | 8, 11 | 10, 11, 12 | 8, 12 | 11, 12 | 11, 12 | 8, 12 | 11, 11 | 11, 11 |
| D16S539 | 11, 13 | 9, 10 | 10, 10 | 9, 9 | 9, 10 | 10. 12 | 11, 12 | 9, 12 | 10, 10 | 12, 12 | 9, 12 | 9, 12 |
| D18S51 | 15, 15 | 13, 15 | 15, 16 | 19, 19 | 13, 13 | 12, 16 | 19, 19 | 13, 16 | 16, 16 | 13, 19 | 15, 20 | 15, 20 |
| D19S433 | 10, 11 | 14, 14 | 14, 14 | 14.2, 14.2 | 13.2, 15.2 | 14, 16.2 | 12.2, 14 | 15, 15.2 | 13, 14 | 13, 13 | 13, 14 | 13, 14 |
| D21S11 | 15, 15.2 | 29, 30 | 28, 31 | 28, 30 | 29, 30 | 28, 34.2 | 29, 31 | 31, 32 | 28.2, 29 | 30, 31.2 | 29, 30 | 29, 30 |
| D2S1338 | 17, 23 | 19, 23 | 24, 24 | 19, 24 | 18, 22 | 17, 17 | 17, 17 | 21, 23 | 17, 27 | 19, 20 | 21, 26 | 21, 26, 27 |
| D3S1358 | 15,18 | 15, 16 | 15, 15 | 14, 15 | 15, 16 | 14, 15, 16 | 15, 15 | 16, 17 | 15, 15 | 15, 17 | 16, 17 | 16, 17 |
| D5S818 | 11, 11 | 12, 12 | 13, 13 | 10, 10 | 11, 12 | 12, 13 | 11, 12 | 11, 11 | 12, 12 | 10, 14 | 10, 13 | 10, 13 |
| D7S820 | 10, 11 | 11, 11 | 10, 11 | 11, 12 | 10, 12 | 12, 12 | 9, 10 | 8, 11 | 10, 11 | 11, 11 | 11, 11 | 11, 11 |
| D8S1179 | 14, 14 | 15, 16 | 10, 15 | 15, 16 | 13, 13 | 13, 16 | 10, 10 | 14, 14 | 10, 13 | 12, 14 | 11, 14 | 11, 14 |
| FGA | 21, 21 | 25, 16 | 21, 24 | 19, 23 | 21, 24 | 20, 22 | 21, 23 | 21, 23 | 22, 24 | 19, 22 | 20, 23 | 20, 23 |
| THO1 | 9.3, 9.3 | 6, 7 | 7, 9 | 9, 9.3 | 7, 7 | 7, 9.3 | 6, 9.3 | 7, 9 | 6, 9 | 6, 6 | 9, 9.3 | 9, 9.3 |
| TPOX | 8, 12 | 11, 11 | 8, 8 | 8, 8 | 9, 11 | 8, 11 | 8, 12 | 8, 11 | 8, 8 | 11, 11 | 11, 11 | 11, 11 |
| vWA | 16, 17 | 17, 17 | 15, 16 | 18, 18 | 17, 18 | 16, 17, 18 | 14, 17 | 14, 18 | 14, 16 | 14, 16 | 19, 20, 21 | 19, 20, 21 |

**Supplementary Table S1. STR data for OCCC cell lines used in this study.** Our ES-2, RMG-I, OVTOKO, OVSAYO, TOV-21G, OVMANA, KOC-7c and OVISE cell line STR data is congruent with STR data for these lines in the CLASTR 1.4.4 database (https://web.expasy.org/cellosaurus-str-search/). SMOV2, TU-OC-1 and 105C STR profiles were checked and found to be unique. The 105C-T line was generated from nodules excised and dispersed in cell culture from murine xenografting studies to show that the nodules contained 105C cells.
