# Supplementary material for: Characterization of Mutational Status, Spheroid Formation, and Drug Response of a New Genomically-Stable Human Ovarian Clear Cell Carcinoma Cell Line, 105C
Source: Cells. 2020 Nov 3;9(11):2408. doi: 10.3390/cells9112408 (PMC7693681; doi:10.3390/cells9112408)
Supplement: Supplementary file 1 [file cells-09-02408-s001.zip › revised Supplementary Figure S3.pptx]

## Slide 1
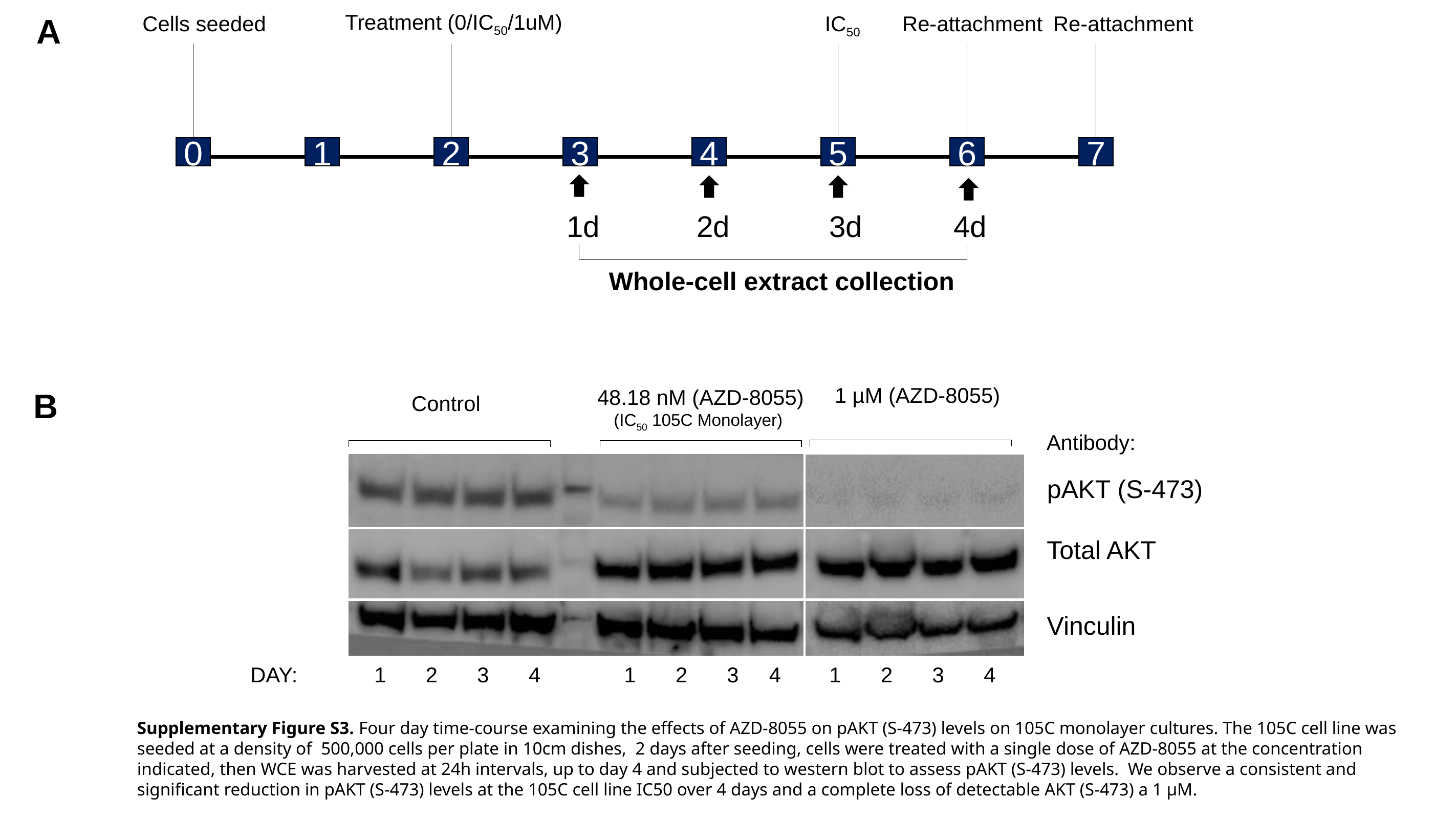

Treatment (0/IC50/1uM)
A
Cells seeded
IC50
Re-attachment
Re-attachment
0
1
2
3
4
5
6
7
1d
2d
3d
4d
Whole-cell extract collection
1 µM (AZD-8055)
B
48.18 nM (AZD-8055)
(IC50 105C Monolayer)
Control
Antibody:
pAKT (S-473)
Total AKT
Vinculin
DAY:
1
2
3
4
1
2
3
4
1
2
3
4
Supplementary Figure S3. Four day time-course examining the effects of AZD-8055 on pAKT (S-473) levels on 105C monolayer cultures. The 105C cell line was seeded at a density of 500,000 cells per plate in 10cm dishes, 2 days after seeding, cells were treated with a single dose of AZD-8055 at the concentration indicated, then WCE was harvested at 24h intervals, up to day 4 and subjected to western blot to assess pAKT (S-473) levels. We observe a consistent and significant reduction in pAKT (S-473) levels at the 105C cell line IC50 over 4 days and a complete loss of detectable AKT (S-473) a 1 µM.
