# Supplementary material for: Characterization of Mutational Status, Spheroid Formation, and Drug Response of a New Genomically-Stable Human Ovarian Clear Cell Carcinoma Cell Line, 105C
Source: Cells. 2020 Nov 3;9(11):2408. doi: 10.3390/cells9112408 (PMC7693681; doi:10.3390/cells9112408)
Supplement: Supplementary file 1 [file cells-09-02408-s001.zip › revised Supplementary Figure S2.pptx]

## Slide 1
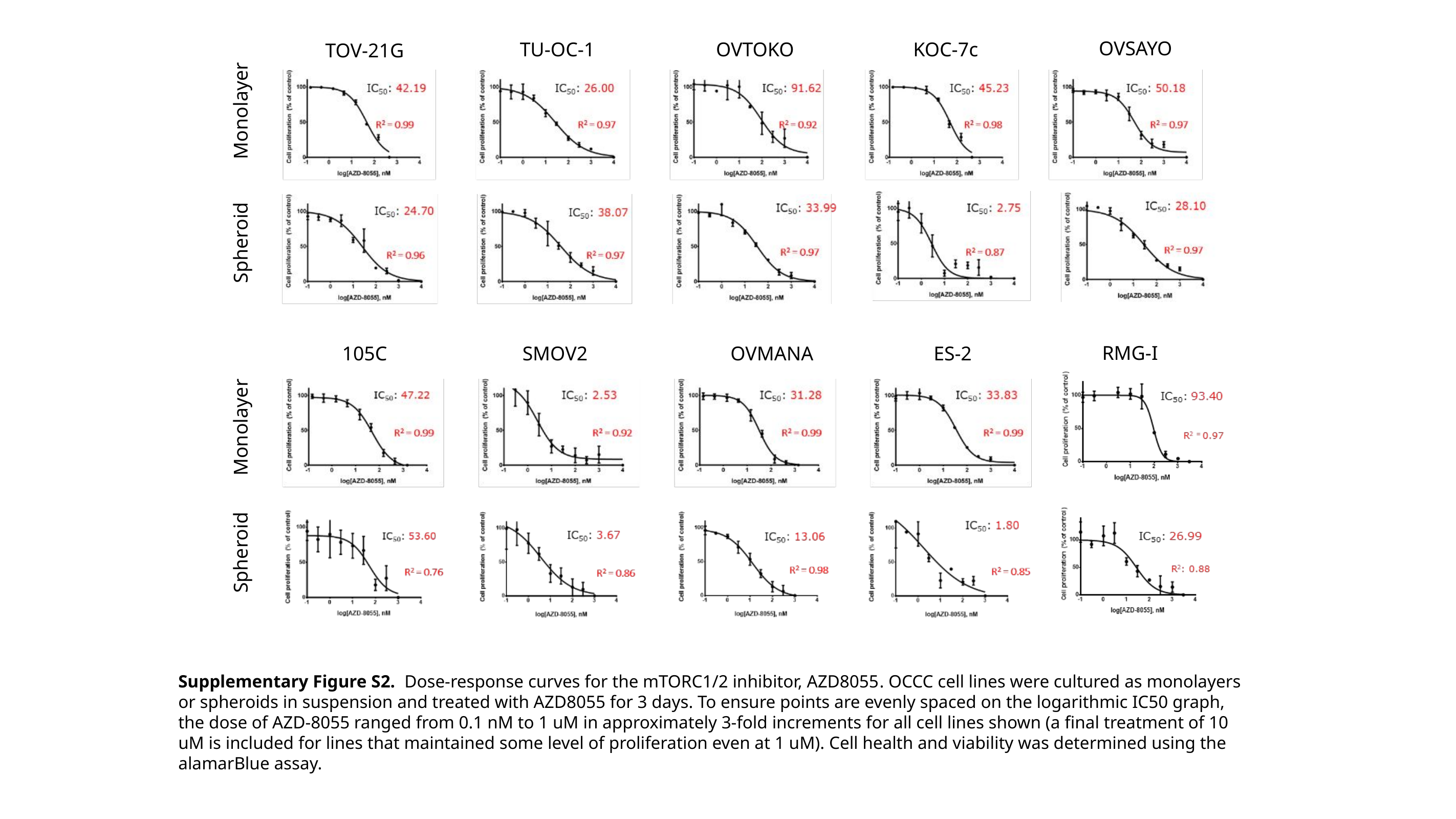

OVSAYO
TU-OC-1
OVTOKO
KOC-7c
TOV-21G
Monolayer
Spheroid
RMG-I
105C
SMOV2
OVMANA
ES-2
Monolayer
Spheroid
Supplementary Figure S2. Dose-response curves for the mTORC1/2 inhibitor, AZD8055. OCCC cell lines were cultured as monolayers or spheroids in suspension and treated with AZD8055 for 3 days. To ensure points are evenly spaced on the logarithmic IC50 graph, the dose of AZD-8055 ranged from 0.1 nM to 1 uM in approximately 3-fold increments for all cell lines shown (a final treatment of 10 uM is included for lines that maintained some level of proliferation even at 1 uM). Cell health and viability was determined using the alamarBlue assay.
