# Supplementary material for: Characterization of Mutational Status, Spheroid Formation, and Drug Response of a New Genomically-Stable Human Ovarian Clear Cell Carcinoma Cell Line, 105C
Source: Cells. 2020 Nov 3;9(11):2408. doi: 10.3390/cells9112408 (PMC7693681; doi:10.3390/cells9112408)
Supplement: Supplementary file 1 [file cells-09-02408-s001.zip › REVISED Supplementary Figure S1.pptx]

## Slide 1
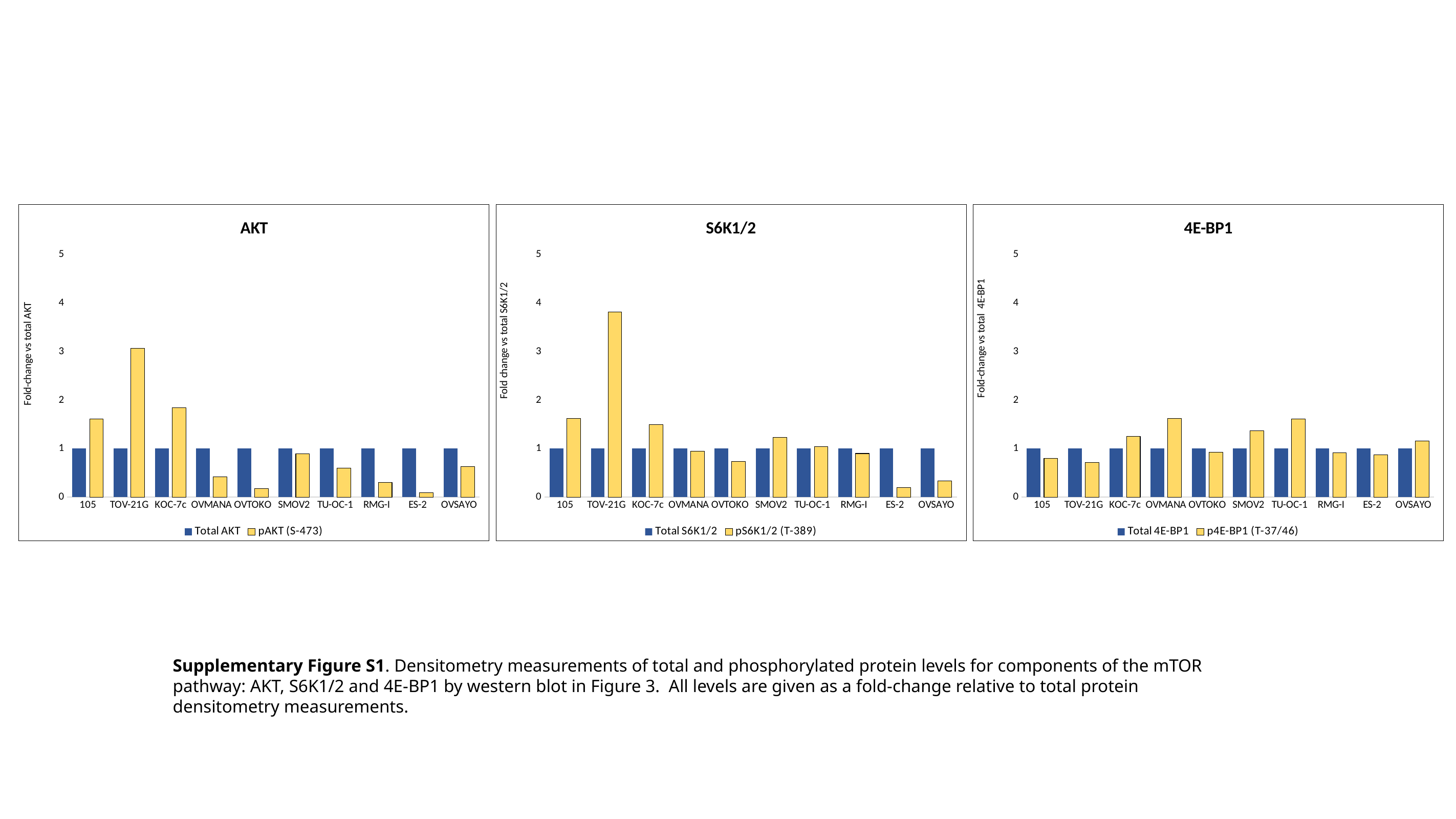

### Chart: AKT
| Category | Total AKT | pAKT (S-473) |
|---|---|---|
| 105 | 1.0 | 1.604857363145721 |
| TOV-21G | 1.0 | 3.063097514340344 |
| KOC-7c | 1.0 | 1.844393592677346 |
| OVMANA | 1.0 | 0.413854351687389 |
| OVTOKO | 1.0 | 0.172348484848485 |
| SMOV2 | 1.0 | 0.888888888888889 |
| TU-OC-1 | 1.0 | 0.598444680098975 |
| RMG-I | 1.0 | 0.305606738615425 |
| ES-2 | 1.0 | 0.0865077414598181 |
| OVSAYO | 1.0 | 0.627012987012987 |
### Chart: S6K1/2
| Category | Total S6K1/2 | pS6K1/2 (T-389) |
|---|---|---|
| 105 | 1.0 | 1.622497616777884 |
| TOV-21G | 1.0 | 3.817596566523605 |
| KOC-7c | 1.0 | 1.497578692493946 |
| OVMANA | 1.0 | 0.941305781490437 |
| OVTOKO | 1.0 | 0.733418367346939 |
| SMOV2 | 1.0 | 1.229418650019508 |
| TU-OC-1 | 1.0 | 1.03835800807537 |
| RMG-I | 1.0 | 0.897208121827411 |
| ES-2 | 1.0 | 0.194647201946472 |
| OVSAYO | 1.0 | 0.329611433675748 |
### Chart: 4E-BP1
| Category | Total 4E-BP1 | p4E-BP1 (T-37/46) |
|---|---|---|
| 105 | 1.0 | 0.793360433604336 |
| TOV-21G | 1.0 | 0.70917396269509 |
| KOC-7c | 1.0 | 1.25278058645096 |
| OVMANA | 1.0 | 1.616596638655462 |
| OVTOKO | 1.0 | 0.923813169984686 |
| SMOV2 | 1.0 | 1.364367046215673 |
| TU-OC-1 | 1.0 | 1.607294317217978 |
| RMG-I | 1.0 | 0.916151925351557 |
| ES-2 | 1.0 | 0.872171801099323 |
| OVSAYO | 1.0 | 1.156905890318213 |Supplementary Figure S1. Densitometry measurements of total and phosphorylated protein levels for components of the mTOR pathway: AKT, S6K1/2 and 4E-BP1 by western blot in Figure 3. All levels are given as a fold-change relative to total protein densitometry measurements.
