# Supplementary material for: Characterization of Mutational Status, Spheroid Formation, and Drug Response of a New Genomically-Stable Human Ovarian Clear Cell Carcinoma Cell Line, 105C
Source: Cells. 2020 Nov 3;9(11):2408. doi: 10.3390/cells9112408 (PMC7693681; doi:10.3390/cells9112408)
Supplement: Supplementary file 1 [file cells-09-02408-s001.zip › revised Supplementary Table S2 - OCCC cell line mutation table.docx]

**Supplementary Table S2. Molecular and phenotypic characteristics of the OCCC cell lines used in this work.**

Gene mutation details were obtained from various published sources and in some cases (indicated by #) a specific mutation is based on our interpretation of chromosomal coordinates provided in (2). The percent copy number alterations (CNA) were generated by our work (*) or obtained from datasets available through cBioPortal ($). Cell line doubling time was generated using an IncuCyte ZOOM system by us (indicated by *) or obtained from the Cellosaurus website (https://web.expansy.org/cellosaurus/). The ARID1A mutation(s) were not specified for the TU-OC-1 cell line by the cited report. WT: wildtype.

| **Cell line** | **Mutation Status** | | | **% CNA** | **Doubling time** |
| --- | --- | --- | --- | --- | --- |
|  | **PIK3CA** | **PTEN** | **ARID1A** |  |  |
| 105C | p.H1047Y p.V344M | p.Y68H, p.K267fs*9 p.Leu316Pro | p.Q505Sfs*117 | 8.1* | 31 h* |
| TOV-21G | p.H1047Y (2) | p.K267Rfs*9 (2)  p.G143Afs*4 (2) | p.Y551Lfs*72 (2, 6) p.Q758Rfs*75 (2, 6) | 6.7^$^ | 25.5 h* |
| KOC-7c | p.K111N (2,5) | p.R233X p.N323Mfs*21# (2,5) | p.M274fs*89# p.Y1324fs*157# p.A1517fs*30 (2)# | 0.9* | 24 h* |
| SMOV-2 | p.H1047L (2) | WT (5) | p.G1740fs*30 (1) | 60.0* | 48.2 h |
| OVTOKO | WT PIK3CA  PIK3R1  p.L449>X (2) | WT (2) | p.E97X (2) p.F2208fs*23 (2)# | 39.9^$^ | 70 h |
| OVMANA | p.E545V (2) | WT (2) | p.S2264X (2, 3) p.Q1332X (2, 3) | 67.8^$^ | 67 h |
| TU-OC-1 | p.E542K (4) | WT (7) | truncating mutation (7) | 61.0* | 26.4 h* |
| RMG-I | WT (3, 7) | WT (3, 7) | WT (3, 7) | 24.1^$^ | 60 h |
| OVSAYO | p.H1047R (3) | WT (3) | p.T294fs*106 (1) | 30.9* | 88 h |
| ES2 | WT (3, 7) | WT (3, 7) | WT (3, 7) | 40.8^$^ | 31 h |

^1^ Our bioinformatic analysis of whole exome data from NCBI BioProject Accession: PRJEB9639 ID: 307433

^2^ Papp E, Hallberg D, Konecny GE, et al. Integrated Genomic, Epigenomic, and Expression Analyses of Ovarian Cancer Cell Lines. Cell Rep. 2018;25(9):2617-2633. doi:10.1016/j.celrep.2018.10.096

^3^ Anglesio MS, Wiegand KC, Melnyk N, et al. Type-specific cell line models for type-specific ovarian cancer research [published correction appears in PLoS One. 2013;8(10). doi:10.1371/annotation/856f0890-9d85-4719-8e54-c27530ac94f4] [published correction appears in PLoS One. 2013;8(9). doi:10.1371/annotation/ffcaf179-872f-470b-8bb6-f06d8ba6d03a]. PLoS One. 2013;8(9):e72162. Published 2013 Sep 4. doi:10.1371/journal.pone.0072162

^4^ Itamochi H, Kato M, Nishimura M, et al. Establishment and characterization of a novel ovarian clear cell adenocarcinoma cell line, TU-OC-1, with a mutation in the PIK3CA gene. Hum Cell. 2013;26(3):121-127. doi:10.1007/s13577-013-0062-y

^5^ Caumanns JJ, van Wijngaarden A, Kol A, et al. Low-dose triple drug combination targeting the PI3K/AKT/mTOR pathway and the MAPK pathway is an effective approach in ovarian clear cell carcinoma. Cancer Lett. 2019;461:102-111. doi:10.1016/j.canlet.2019.07.004

^6^ cBioPortal <https://www.cbioportal.org>

^7^ . Caumanns JJ, Berns K, Wisman GBA, et al. Integrative Kinome Profiling Identifies mTORC1/2 Inhibition as Treatment Strategy in Ovarian Clear Cell Carcinoma. Clin Cancer Res. 2018;24(16):3928-3940. doi:10.1158/1078-0432.CCR-17-3060
